# The older, the better: a comprehensive survey of soil organic carbon under commercial oil palm plantations

**DOI:** 10.1007/s10661-024-13540-y

**Published:** 2024-12-21

**Authors:** Karolina Golicz, Sim Choon Cheak, Suzanne Jacobs, André Große-Stoltenberg, Mojdeh Safaei, Sonoko Bellingrath-Kimura, Lutz Breuer, Ariani Wartenberg

**Affiliations:** 1 Institute for Landscape Ecology and Resources Management (ILR), Research Centre for Biosystems, Land Use and Nutrition (iFZ), Justus Liebig University Giessen, Heinrich-Buff-Ring 26, 35392 Giessen, Germany; 2 SD Guthrie Research, SD Guthrie Berhad, Jalan Pulau Carey, Malaysia; 3 Centre for International Development and Environmental Research (ZEU), Justus Liebig University, Giessen, Germany; 4 Division of Landscape Ecology and Landscape Planning, Institute of Landscape Ecology and Resource Management (ILR), Research Centre for Biosystems, Land Use and Nutrition (iFZ), Justus Liebig University Giessen, Heinrich-Buff-Ring 26, 35392 Giessen, Germany; 5 Leibniz Centre for Agricultural Landscape Research (ZALF), Müncheberg, Germany; 6 Faculty of Life Science, Humboldt University of Berlin (HU), Berlin, Germany

**Keywords:** Soil conditions, Multilevel modelling, GIS-derived regional predictors

## Abstract

Soil conditions of croplands are a frequent topic of scientific research. In contrast, less is known about large-scale commercial plantations of perennial crops such as oil palm. Oil palm is a globally important tropical commodity crop which contributes to both food and energy security due to its exceptional productivity. However, oil palm crops are associated with short lifecycles and high nutrient demands, which may disproportionately affect soil health. With the goal of exploring baseline soil properties in commercial oil palm plantations, we evaluated data from two large-scale soil surveys carried out in 2014/2015 and 2018/2019 across more than 400 fields located throughout Peninsular Malaysia. We examined variation in field-measured soil quality indicators with a focus on soil organic carbon content at three depths (0–15 cm, 15–30 cm, 30–45 cm) and investigated links with spatial covariates, including plantation age. We found SOC contents to be low (1.6–2%) across the sampled locations with limited correlation with spatial predictors employed in soil organic carbon modelling. Furthermore, we found that immature and young mature plantations, which consisted of fields that were re-planted as part of a 20-year-long oil palm rotation, were characterised by significantly lower soil organic carbon content than the mature plantations. This suggests that management practices should target younger oil palm plantations for soil organic conservation measures to increase the overall baseline SOC content, which will subsequently accumulate over the plantation’s lifespan. We further provide recommendations for future soil sampling efforts, which could increase the robustness of collected data and facilitate their use for soil monitoring through modelling approaches involving, for example, digital soil mapping.

**Supplementary Information:**

The online version contains supplementary material available at 10.1007/s10661-024-13540-y.

## Introduction

### Global context: oil palm impacts, sustainable management practices, and soil organic carbon

The cultivation of tropical commodity crops is an important contributor to global land-use change and related land degradation dynamics (e.g. Descals et al., 2021). Of tropical crops, oil palm (*Elaeis guineensis*) has the highest production volume and trade (Rahman et al., 2021), with global cultivation area occupying 21 million ha (Meijaard et al., 2020) and continuing to rapidly increase (Petrenko et al. 2016). Over two-thirds of global palm oil is produced in Malaysia and Indonesia (Tapia et al., 2021). Conventional oil palm cultivation has been associated with widespread environmental degradation, including biodiversity loss, soil quality decline, and greenhouse gas emissions from initial land clearing and intensive crop management practices (Frazão et al., 2014; Tapia et al., 2021; Yahya et al., 2010). However, the latter practices play a key role in determining the ecological impact of crop cultivation, including soil health and carbon sequestration (Rahman et al., 2021).

One of the most important and universal indicators to monitor the impacts of land management on agroecosystems is the soil organic carbon (SOC) stock, i.e. the amount of organic carbon measured in units of mass that is present in a given area (Lorenz et al., 2019; Lorenz and Lal, 2016). High SOC content has been linked to improved ecosystem health and resilience through processes relating to soil biodiversity and other soil health indicators such as aggregate stability, infiltration rates, and water retention capacity (Lorenz et al., 2019; Paustian et al., 2019). Prioritising the sustainable intensification of existing plantations through management practices that target improved soil fertility and increased SOC content may thus be a promising approach to ensure the sustainability of already-established fields and mitigate further ecosystem degradation (Frazão et al., 2014; Rahman et al., 2021). For example, several studies have shown that amending soils through the incorporation of the frond and harvest residues can contribute to increased SOC storage over time in oil palm plantations in Brazil, Malaysia, and Indonesia, with either neutral or positive impacts on yields (e.g. Frazão et al., 2014; Haron et al., 1998; Rahman et al., 2021). The inclusion of ‘tree islands’ in conventionally managed oil palm cultivation landscapes has further been shown to contribute to improved biodiversity and ecosystem functioning, including soil fertility—without leading to measurable yield decreases (Zemp et al., 2023). However, to assess the impact of these promising land management activities on soil properties, especially SOC, sound soil sampling and monitoring plans must be put in place.

### Soil sampling for long-term monitoring: state of the art and knowledge gaps

Addressing the spatial variability of soil properties (e.g. Lin et al., 2005), including SOC, constitutes an important challenge to developing accurate estimates of soil characteristics, especially at large spatial scales. Soil sampling is often carried out in a grid. This allows for the application of traditional geospatial statistical methods (e.g. ordinary kriging) to interpolate the distribution of soil properties; however, given that the method requires taking a high density of samples, it is particularly relevant for smaller land parcels (Pouladi et al., 2019). In addition, soil sampling design is generally informed by consideration of parameters known to determine soil distribution and its properties—including, for instance, geomorphology or vegetation cover (McBratney et al., 2003). Consideration of these parameters may reduce the spatial variability of samples. For example, this can be done by carrying out sampling on a stratified a priori basis, using strata such as soil parent material, or by standardising sampling within topographic units (Turner & Lambert, 2000). Modern best practice encourages stratification by coarse geospatial covariates, taking advantage of the widespread availability of environmental and land management data, to augment, e.g. SOC predictions at the field (e.g. Wang et al., 2023) and regional scales (e.g. Brus, 2019). The application of advanced sampling strategies such as conditioned Latin hypercube (using a model-based approach to optimise sampling in selected strata, detailed in, e.g. Brus, 2019) can result in a more targeted and cost-effective approach to collecting field data (Bettigole et al., 2023). These techniques are well established in the scientific community; however, more awareness needs to be brought to their utility in the private sector.

In addition to spatial aspects of the sampling design, considering temporal resolution by developing robust long-term soil monitoring strategies is paramount to understand the impacts of current production systems and to promote sustainable land management in the future (Richter et al., 2007). Turner and Lambert (2000) detail four techniques for long-term soil monitoring in annual cropland and tree plantations, particularly, to assess SOC sequestration, i.e. (1) paired sites to compare soil properties with previous land-use or successive rotations (e.g. Rahman et al., 2021), (2) chronosequence studies with investigations of varied age stands characterised by similar site conditions and management (e.g. Zhijun et al., 2018), (3) multiple resampling of the same soils across many years (e.g. Cerdà et al., 2021), and (4) process and modelling studies which estimate potential inputs and losses within the system. However, such long-term soil experiments often rely on intensive sampling at small spatial scales. While they offer valuable insights on long-term soil processes, they are also limited in scope and generalisability. In response, scientists are increasingly leaning on methods which combine direct measurements, modelling, and remote sensing (‘digital soil mapping’) to estimate and model SOC stocks, as this integrative approach holds particular promise in terms of balancing cost-effectiveness and accuracy (Mandal et al., 2022; Minasny & McBratney, 2016).

Ultimately, the question of robust and long-term sampling strategies is particularly relevant as the remote monitoring of carbon budgets across supply chains becomes more widespread. In palm oil plantations, exploring this topic has the potential to contribute to improving the sustainability of the industry. Indeed, the necessity to develop good-practice monitoring approaches has been recognised by the Roundtable on Sustainable Palm Oil (Nunes et al., 2017). In this context, defining concrete goals linked to soil data collection, e.g. application of environmental predictor-dependent statistical methods for SOC mapping and remote monitoring or development of an empirical process-based model to identify drivers of SOC accumulation, will aid the selection of suitable sampling and monitoring strategies. In Malaysia, researchers have documented above-ground biomass and carbon densities associated with palm oil cultivation areas using remote sensing approaches such as the integration of satellite imagery and machine learning (Shaharum et al., 2020) and the application of LiDAR technology (Nunes et al., 2017). However, large-scale assessments of the magnitude and spatial distribution of below-ground carbon storage and SOC stock change dynamics in oil palm–dominated landscapes remain lacking.

### Novelty, impacts, and research aims

Here, we capitalise on an opportunity to conduct an analysis of baseline soil survey data in commercial oil palm plantations in west Malaysia. We analysed data originating from two consecutive and large-scale soil surveys, conducted across more than 400 fields across peninsular Malaysia, covering an area of 30,000 ha. We applied a mixed-model analysis approach to evaluate variation in field-measured soil quality indicator i.e., the SOC content and to further investigate links with a set of nine spatially explicit covariates, including management-based, pedological, and environmental variables. Our research aims are threefold:

i. Describe baseline soil conditions in a commercial oil palm plantation based on two large-scale soil sampling campaigns;
ii. Identify significant management and environmental drivers of soil properties with special reference to SOC content; and
iii. Develop recommendations for follow-up long-term soil monitoring strategies.

The research presented here may contribute to the development of practical strategies for long-term monitoring of SOC change, with implications relevant to soil management across the region. Specifically, we aim to generate recommendations designed to focus on SOC stocks, inform assessment of land management impacts on C storage sequestration rates, and facilitate future digital soil mapping efforts.

## Methodology

### Study area

**Fig. 1:**
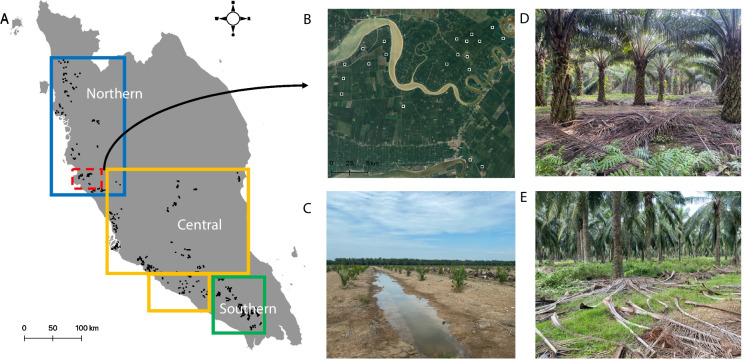
Sampling locations (denoted by black squares) and designated management regions in peninsular Malaysia (**A**), bird’s eye image of an exemplary subset of sampling locations within the estate marked in red (**B**; Google, n.d.) and an immature oil palm field (**C**), a mature oil palm field (**D**), and an old oil palm field (prior to reestablishment, **E**). Photos by Karolina Golicz

The study area covers 30,000 ha of commercial oil palm plantations managed by SD Guthrie Berhad located in west (peninsular) Malaysia (Fig. 1; UTM zone 50 N). The climate of the studied region is classified as humid tropical with a mean annual temperature of 25.4 °C and mean annual precipitation (MAP) of 3085 mm (The World Bank Group 2021). The topography is characterised by low elevation (range 4 to 116 m with slope < 4.2°). Two soil groups, i.e. Fluvisols and Acrisols (World Reference Base for Soil Resources, 2023), are predominant with texture ranging from loamy (north-western coastline) to sandy clay (south-western coastline). Previous land cover history (e.g. rubber plantations, smallholder farms, primary or secondary rainforest) of individual fields is unknown. Three regions, i.e. northern, central, and southern, were delineated by SD Guthrie Berhad and represent designated management zones.

At the time of sampling, the plantations had a mean (rotation) age of 13 (± 6) years with the youngest plantation re-planted in 2018 and the oldest in 1989 (rotation period for oil palm is ca. 20 years). The planting density is between 128 and 160 stems ha^−1^ on hilly and flat terrain, respectively. The fields are conventionally managed via the application of inorganic fertiliser (35 kg ha^−1^ of nitrogen, phosphorus, and potassium (NPK) fertiliser). Fertiliser application is carried out twice a year, and the rates differ depending on the growth stage and the palms’ fertility status. Additional management activities comprise harvest (every 2 weeks for mature bunches), pruning, and deposition of pruning and harvest residues between oil palms.

### Soil sampling and chemical analysis

Soil sampling was carried out in 2014/2015 and 2018/2019 by SD Guthrie Berhad. The first sampling campaign (C1) was carried out in the northern and southern management regions and comprised 245 samples. The second sampling campaign was carried out in the northern, central, and southern management regions (C2) and comprised 468 soil cores. Seventy-six C1 sampling locations were resampled during C2. Out of these, 39 locations (15 in the north and 24 in the south) include a full set of baseline soil data.

Soil cores were collected along a 100 m × 100 m grid, and 50–60 cores were composited to obtain final samples: in effect, each single composite sample thus represents 50–60 ha. The cores were collected down to a depth of 45 cm with samples divided into three increments (0–15 cm, 15–30 cm, and 30–45 cm), resulting in a total of *n* = 2576 data points. The maximum depth of 45 cm was selected as per SD Guthrie Berhad’s internal soil sampling protocol, i.e. the industrial standard. The auger size was set at 15 cm, and three depths were sampled. Bulk density measurements were not collected as part of either sampling campaign. A lack of bulk density measurements precluded any statistical inference regarding changes to SOC stocks due to the potential for errors in the estimation of the soil profile length, which results from changes to the bulk density values (Fowler et al., 2023). Thus, only trends of SOC change between C1 and C2 were presented.

Soil samples from C1 to C2 were air-dried, sieved, and analysed for SOC content, pH, and cation exchange capacity (CEC) following in-house operating procedures based on global standards. The SOC content (100 mg sub-samples) was determined via the dry combustion method, using an Elementar SoliTOC apparatus (Elementar, Germany). The soil pH (10 g sub-samples; 2 mm sieved, over-dried) was measured in a 1:2.5 soil:deionised water suspension and analysed with a glass pH electrode (Consort). The soil cation exchange capacity (10 g sub-samples; 2 mm sieved, over-dried) was measured using the ammonium acetate method (Sharifuddin et al., 1990). Each depth increment was analysed separately.

### Data management

We checked the full dataset resulting from C1 to C2 (*n* = 2576 data points) for duplicates, blank, or inconsistent data entries, which were subsequently removed, reducing the dataset to a final *n* = 2139 data points. Following this initial screening, the dataset was split to account for the difference between mineral and organic soils. Six samples fit the definition for organic soils with SOC > 12% (Calvo et al., 2006) and were excluded from the subsequent analysis (reason: dataset was too limited for statistical analysis). Data entries with missing coordinates (*n* = 756) were further excluded, after the calculation of descriptive statistics. The remaining (*n* = 1380) dataset was split according to three depth increments, which resulted in *n* = 105 georeferenced records for each depth increment for the 2014/2015 and 353 records for the 2018/2019 sampling campaigns. Additionally, samples with incomplete records, i.e. missing CEC values, were removed (*n* = 147 in total). The depth increments were analysed separately.

The selection of covariates for modelling of soil properties, with special reference to SOC content, followed the SCORPAN framework (based on seven soil forming factors) and the scale-dependent hierarchy of drivers and indicators proposed by Wiesmeier et al. (2019) with the scale set at ‘regional’. The final set of predictors was further refined based on recent literature (Cahyana et al., 2023; Guo et al., 2015; Sakhaee et al., 2022; Sothe et al., 2022) and understanding of the local site conditions.

The plot-scale covariates involved parameters measured as part of the soil sampling campaign, i.e. pH, CEC, SOC content, and plantation age, i.e. time since (re)planting. Plantation age was classified into five distinct categories, i.e. 0–3 years (immature), 4–6 (young mature), 7–12 (prime), 13–20 (mature), and over 20 (old) (Fig. 1C–E) as per description provided by SD Guthrie Research. We note that the exact direction in which the sample collection was carried out across 50 ha was unknown, impeding the resampling to a resolution of less than 50 ha, i.e. multiple samples were bulked together and the coordinates of individual samples making up the bulk sample were not recorded. However, the resolution of regional-scale environmental covariates was set to 250 m^2^ instead of 50 ha because they were found to better correlate with the response variable. The topographical predictors were derived from FABDEM V1-0 (Hawker & Neal, 2021) with a 30 m resolution (re-projected to EPSG 3375, i.e. Geodetic Datum of Malaysia 2000 using meters as units using Whitebox tools (vers. 1.0.9 in QGIS)). The remote sensing products, i.e. mean and median NDVI and EVI, were derived from the MODIS. The distances to the coastline and the nearest waterway were calculated with the *dist2Line* function in the *geosphere* R package. The preprocessing of raster and shapefile layers used to derive the predictors was carried out in QGIS (ver. 3.32) and R (ver. 4.2).

### Statistical analysis

Data exploration followed an 8-step protocol developed by Zuur et al. (2010). Response variables (SOC content, pH, and CEC) were investigated in reference to outliers, homogeneity, normality, and spatial independence. The covariates (explanatory variables, Table 1) were checked for the presence of outliers, collinearity, and the strength of the relationship with the response variables. An initial screening showed that geographic information system (GIS)–derived environmental covariates (regional scale) did not correlate strongly with the measured soil properties (for details, see the R script titled Data Preprocessing; subsection: Data Exploration—Relationships between variables); subsequent analyses thus focused on plot-scale variables. Interactions were visually inspected through the application of co-plots. Multicollinear explanatory variables were identified via scatterplots and excluded from further analysis (criteria for exclusion: correlation coefficient > 0.5 between variable pairs).

**Table 1:** Covariates selected for modelling of soil properties with special reference to SOC content (%). Raster resolution: 250 m; projection for topographical covariates: EPSG 3375; projection for the remaining covariates: EPSG 4326

Scale		Covariate	Source
Plot		pH	SD Guthrie Research database
		Age (years)	SD Guthrie Research database
		CEC (cmol kg^−1^)	SD Guthrie Research database
		SOC content (%)	SD Guthrie Research database
Regional	Climate	MAP (mm)	https://worldclim.org
		Northern, central, and southern regions (Fig. 1)	SD Guthrie Research database
	Vegetation	NDVI, EVI (mean and median), difference between NDVI and EVI	https://modis.gsfc.nasa.gov
	Topography	Elevation (m), aspect, slope (°), TWI, terrain curvature	https://gee-community-catalog.org/projects/fabdem/
		Distance to the nearest waterway	https://www.hotosm.org
		Distance to the coast	https://geodata.lib.berkeley.edu
	Parent material	Geological map of peninsular Malaysia	https://www.usgs.gov
		Presence and distance to peatlands	https://www.globalforestwatch.org

Abbreviations:* CEC* cation exchange capacity, *SOC* soil organic carbon, *MAP* mean annual precipitation, *NDVI* normalised difference vegetation index, *EVI* enhanced vegetation index, *TWI* topographical wetness index

Further selection of explanatory variables for modelling of drivers of soil properties was carried out by means of backward selection using the ‘base model’ (a mixed effect model with ‘estate’ as random effects and no covariates) for comparison with a ‘full model’ (with the same random structure as the ‘base’ model) containing a set of 13 non-collinear predictors, which were scaled prior to the analysis. To achieve a near-normal distribution of the residuals, the response variables were log-transformed, and the *varIdent* function was included in the model to alleviate the heteroscedasticity of the residuals for the ‘age class’ variable. Eight variables were retained in addition to the SOC content: management-based variables (region, sampling campaign, age class, pH), pedological variables (CEC, geological unit), and an environmental variable (distance to coastline) for further analysis. The median NDVI was indicated as a significant explanatory variable based on the Akaike Information Criterion (AIC) score but was dropped from the analysis upon inspection of the residual *vs*. covariate plot (post-transformation).

Summary statistics for soil parameters in respect to regions and sampling campaigns were generated, and piecewise structural equation models (SEM) were employed to detect the direct and indirect effects of selected management-based, pedological, and environmental factors on the soil properties. The SEM models were fitted separately for each soil depth using the piecewiseSEM R package (Lefcheck, 2016). Models with the fewest variables (minimal adequate models), the lowest AIC score, and the highest chi-square and *p*-value were selected as final models of drivers of soil properties. Finally, a generalised least square model with a rational correlation structure to account for spatial dependencies (Golicz et al., 2023), followed by a Tukey post-hoc test (by means of *multcomp* R package), was used to examine significant differences in the SOC content in regard to plantation age for different regions and depths to better visualise the relationships identified via SEM. The statistical analysis was carried out using R (ver. 4.2).

## Results

### Baseline soil conditions in commercial oil palm plantations

Three soil properties were measured at SD plantations: pH, CEC (ccmol kg^−1^), and SOC content (%) (Table 2). Soil pH was slightly acidic with limited variation in average pH down the soil profile and across the sampling regions. On average, CEC decreased slightly with soil depth (0.22-unit change). We found consistent regional variation in CEC values across both sampling periods: values in the northern region were the highest (C1 mean, 15.60 ± 10.6, and C2 mean, 12.60 ± 9.36); values in the southern region were the lowest (C1 mean, 5.02 ± 3.07, and C2 mean, 5.07 ± 3.74).

**Table 2:** Mean (± SD) soil pH ( −), cation exchange capacity (CEC) (cmol kg^−1^), and soil organic carbon content (SOC) (%) of mineral soils sampled in the periods of 2014–2015 and 2018–2019. All sites (including non-georeferenced samples and samples with missing CEC values) were considered for summary statistics

Region	Depth	Sampling campaign					
		2014/2015			2018/2019		
		pH	CEC (cmol kg^−1^)	SOC (%)	pH	CEC (cmol kg^−1^)	SOC (%)
Northern	0–15 cm	5.0 ± 1.5	15.80 ± 10.70	1.72 ± 1.29	4.4 ± 0.6	12.70 ± 9.56	1.61 ± 1.06
	15–30 cm	4.8 ± 1.7	15.30 ± 10.60	1.41 ± 1.24	4.3 ± 0.7	12.50 ± 9.38	1.31 ± 1.02
	30–45 cm	4.7 ± 1.7	15.60 ± 10.50	1.15 ± 1.17	4.3 ± 0.7	12.60 ± 9.20	1.06 ± 0.88
Central	0–15 cm	-	-	-	4.4 ± 0.6	9.28 ± 7.19	1.55 ± 1.20
	15–30 cm	-	-	-	4.3 ± 0.6	9.05 ± 7.32	1.14 ± 0.92
	30–45 cm	-	-	-	4.2 ± 0.6	9.10 ± 7.59	0.93 ± 0.81
Southern	0–15 cm	5.0 ± 1.1	5.54 ± 3.29	2.05 ± 1.45	4.6 ± 0.5	5.48 ± 3.76	1.74 ± 1.10
	15–30 cm	4.9 ± 1.0	4.79 ± 3.15	1.53 ± 1.42	4.5 ± 0.4	5.02 ± 3.80	1.23 ± 1.14
	30–45 cm	4.8 ± 1.0	4.71 ± 2.70	1.12 ± 0.67	4.5 ± 0.4	4.72 ± 3.66	0.96 ± 1.17

Soil OC content decreased with soil depth (range of mean unit change every 15 cm: 0.25 to 0.51). However, the SOC variation among regions differed across the two sampling periods. For C1 data, values were highest in the southern region (C1 mean, 1.56 ± 1.29), whereas for C2 data, values were highest in the northern region (C2 mean, 1.33 ± 1.18) (Table 2).

### Trends in soil conditions for sites sampled in 2014/2015 and resampled in 2018/2019

**Fig. 2:**
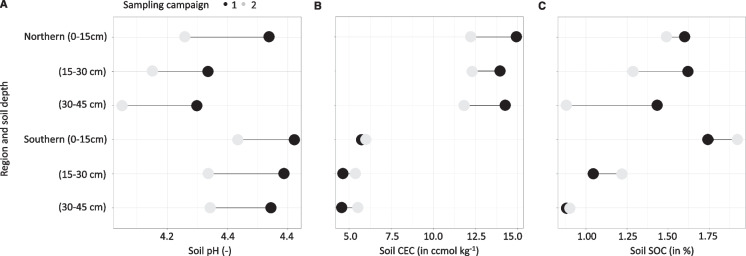
**A**–**C** A change in mean soil pH (**A**), cation exchange capacity (**B**), and organic carbon (**C**) between 2014/2015 (1) and 2018/2019 (2) sampling campaigns in relation to the sampling region (northern: *n* = 14 and southern: *n* = 24) and soil depth. No field was re-planted (as part of the 20-year-long rotation) between the two time points. Note that results for the central region cannot be shown as data for 2014/2015 are not available

Between 2014/2015 and 2018/2019, mean soil pH values decreased in both the northera and southern regions and across all three soil depths: in the north, we observed decreases of 0.3, 0.2, and 0.2, and in the south by 0.2, 0.2, and 0.2, in the topsoil (0–15 cm), subsoil (15–30 cm), and deep soil (30–45 cm) layers, respectively (Fig. 2A). We observed regional differences in mean CEC variation: mean CEC decreased in the north by 2.55, 1.67, and 2.54 cmol kg^−1^ and increased in the south by 0.20, 0.57, and 0.83 cmol kg^−1^ in the top-, sub-, and deep soil, respectively (Fig. 2B). Observed variation in SOC contents from C1 to C2 also showed regional differences: SOC decreased in the north by 0.10, 0.32, and − 0.16% and increased in the south by 0.15, 0.16, and 0.01% along the soil profile (Fig. 2C).

### Environmental and management-based drivers of soil properties

**Fig. 3:**
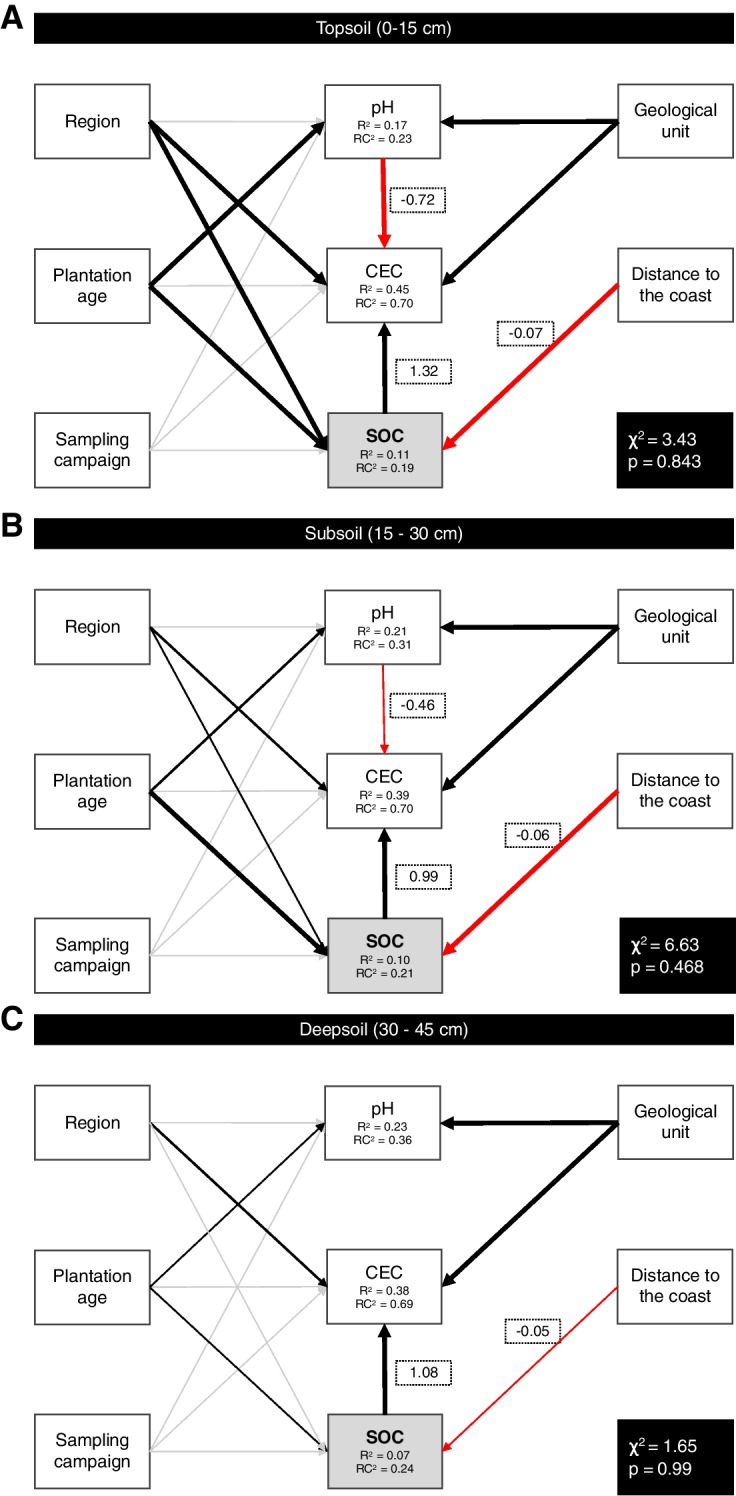
**A**–**C** Structural equation models (SEM) exploring the effects of management-based (region, plantation age, and sampling campaign), pedological (geological unit), and environmental factors (distance to the coast) on topsoil (**A**), subsoil (**B**), and deep soil (**C**) soil properties of Malaysian oil palm plantations. Boxes represent measured variables. Arrows represent unidirectional relationships among variables. Black arrows denote positive relationships, and red arrows negative ones. Arrows for non-significant paths (*p* > 0.05) are light grey. The thickness of the significant paths was scaled based on the magnitude of the standardised regression coefficient (for continuous variables) and the *p*-values (for categorical variables), given in the associated box. The *R*^2^ values of component models are given in the boxes of response variables. The associated chi-square and *p*-value for the entire model are given in black boxes

The SEM models detected significant positive indirect effects of region and plantation age on CEC via SOC content and soil pH (Fig. 3A–C). Geological strata had a significant direct effect on soil pH and CEC but not on the SOC content. The SEM models detected a positive direct effect of plantation age on the SOC content across the entire soil profile, whereas the distance to the coast was found to have a direct negative effect on SOC content. No direct or indirect effects of the sampling campaign on the soil properties were detected at any depth.

### Impact of plantation age on SOC content

**Fig. 4:**
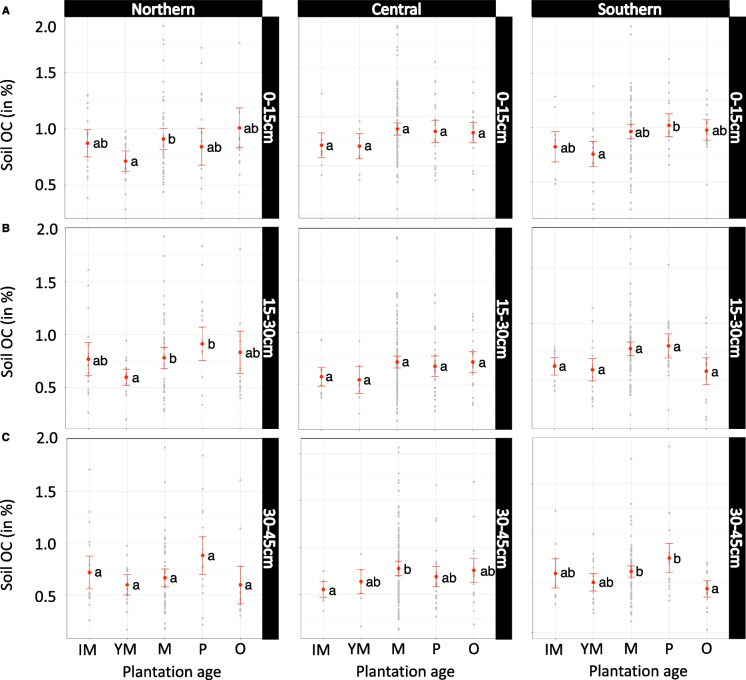
Soil organic carbon content (log-transformed) in response to age class of the plantation in the northern, central, and southern regions at three depth layers: 0–15 cm (**A**), 15–30 cm (**B**), and 30–45 cm (**C**). The letters denote statistically significant differences (groups that do not share a letter are statistically different). Raw data (log-transformed) in grey; fitted means (± 95% confidence interval) in red. Abbreviations: IM, immature (0–3 years); YM, young mature (4–6 years); P, prime (7–12 years); M, mature (13–20 years); O, old (over 20 years)

In the northern region, the mean topsoil (0–15 cm) and subsoil (15–30 cm) SOC content in ‘young mature’ plantations was significantly lower (*p* < 0.03) in comparison to ‘mature’ plantations (Fig. 4A, B). No difference was noted for deepsoil (Fig. 4C). In the central region, no significant differences among plantations were found (Fig. 4A, B). In deepsoil, the ‘immature’ plantations had significantly lower SOC content relative to ‘mature’ plantations (*p* < 0.05) (Fig. 4C). In the southern region, the mean SOC content of ‘young mature’ plantations was significantly lower than ‘prime age’ plantation (*p* < 0.05) (Fig. 4A). The deepsoil content of SOC recorded for ‘old’ plantations was significantly lower than that of ‘prime age’ plantations (*p* < 0.01) (Fig. 4C).

## Discussion

### Insights from baseline soil data at oil palm plantations

Baseline soil conditions indicated regional differences for the measured parameters, in particular for CEC and SOC content. Notably, we observed that increases in soil CEC were associated with increasing SOC content (Fig. 2B, C; *R*^2^ = 0.45; Supplementary Material: Fig. S1; a detailed data exploration protocol is documented in the *Data preprocessing* script made available alongside this manuscript), which was previously linked to highly weathered tropical soils (Soares & Alleoni, 2008). We found lower mean SOC contents across the plantations (topsoil, 1.6–2%) compared to values recorded for East Malaysian forests (topsoil, 2.5%), confirming prior observations of SOC declines after the conversion from forest to oil palm (Rahman et al., 2018). Our findings were consistent with previous values recorded for East Malaysian oil palm plantations (1.5–2.3%) (Rahman et al., 2018). Furthermore, we found indications of decreasing SOC content in the north and increasing SOC content in the south between the two sampling periods, potentially due to land management effects (explained in more detail in the following section).

During data processing and subsequent outlier identification, areas with very high pH (> 10) and high SOC content (> 7.6%) were noted (Supplementary Material: Fig. S2A-B). The presence of exceptionally basic soils among our samples highlights the importance of soil testing for targeted fertiliser management, which may promote nutrient use efficiency, e.g. via remediation and phosphate immobilisation prior to fertiliser application (Ferrarezi et al., 2022). For high SOC content sites, we examined relationships with potential drivers of elevated SOC, e.g. peatland presence/absence and distance to the nearest peatland area (map source: Global Forest Watch, 2014). However, we found no evidence linking these drivers with high SOC content; this may be due to the limited accuracy of the global peatland maps used in our analyses (Minasny et al., 2023). Other potential factors contributing to our results may have been related to missing or incomplete data regarding (1) soil texture, which is one of the strongest determinants of soil carbon storage capacity and its sequestration potential (Wiesmeier et al., 2019); (2) plantation rotation cycles (the number of times each field was re-planted); and (3) historical land use. For instance, we expect that differences in management practices in former flooded rice paddies versus rubber plantations would contribute to variable baseline soil conditions following conversion to oil palm. Ultimately, large-scale perennial crop plantations provide a unique setting for investigating the trajectory of change in soil conditions for different historical land use types because the same vegetation cover limits variability of inputs; provided the data on former land use is reliably collected, georeferenced, and stored.

At large spatial scales, e.g. the 30,000 ha covered in our analysis, costs of laboratory analysis become prohibitive (Smith et al., 2020) and are often mitigated by the use of soil sample bulking. However, sample bulking likely contributes to uncertainties in field-scale assessments, e.g. due to high spatial variability of soil properties and signal-to-noise issues (Paustian et al., 2019). In such instances, modern technologies such as the ICRAF-ISRIC VNIR spectral library of world soils (Viscarra Rossel et al., 2016) developed by means of novel tools, i.e. benchtop and portable spectrometers (Nocita et al., 2015), can be employed by the industry for sensing soil quality to decrease the costs of long-term monitoring and improve the overall land management.

### Drivers of soil organic carbon concentration in oil palm plantations

Soil organic carbon content serves as an important performance indicator for sustainable agricultural management practices (Grahmann et al., 2023) and as a proxy measure for land degradation (Lorenz et al., 2019). In oil palm production, it is of particular because of a strong positive linear relationship with fresh fruit bunch yield (*R*^2^ = 0.85; Rahman et al., 2021). Numerous studies have investigated environmental drivers of SOC accumulation (summarised in, e.g. Wiesmeier et al., 2019). However, we note that the expected relationships with commonly investigated environmental variables (e.g. elevation, topographical wetness index) affecting SOC accumulation were poor for our study. This may be linked to data resolution issues across our datasets and exacerbated by SOC sample compositing across a large area (50 ha), which likely reduced the accuracy of our GIS-derived covariates.

Nevertheless, we identified several key drivers of SOC content through our analysis: *distance to coast*, *region*, and *age of the plantation*. *Distance to coast* (mean, 30.5 km; max, 115.8 km; and min, 0.34 km) had a negative impact on the SOC content. This parameter combined effects of a number of terrain and climatic characteristics, e.g. in Malaysia, plantations further away from the coast have higher elevation and thus are more likely to have more variable topography, cooler climate, and altered rainfall patterns. Hence, *distance to coast* acts as a better predictor than individual covariates such as elevation, which have a coarse spatial resolution (250 × 250 m) in this study.

In terms of *regional differences*, we found higher SOC contents in the southern region. This was an unexpected finding, as the fields in the southern sampling region are characterised by lower planting densities due to steeper slopes and low CEC values often associated with limited land productivity (Takoutsing et al., 2016)—factors that can also negatively impact carbon sequestration rates. Differences in regional management practices should be investigated in more detail. For instance, existing soil conservation measures such as slope stabilisation via the planting of annual vegetation for soil cover, or soil redistribution via terracing (De Blécourt et al., 2014), were more commonly practiced in the southern region of our study area (pers. communication with an on-site agronomist). Further investigation of how these practices may have influenced soil property outcomes may inform the identification of specific measures that can be scaled up for better soil management.

Finally, we identified significant effects of *plantation age* on SOC content. In their investigation of the impacts of plantation age (i.e. time since conversion) and maturity stage (i.e. number of rotation cycles) on SOC content, Rahman et al. (2018) observed initial sharp declines in SOC content (a decline of 40% relative to the reference) after land conversion from the forest. The authors also observe that stocks appear to build back over time, with similar SOC stocks in the oldest (49-year-old) plantations and original reference forest plots. Our observation of SOC increases with increased oil palm maturity echoes the findings of Rahman et al. (2018). However, we also noted significantly lower SOC content in immature, young mature, and old fields in comparison to mature and prime age fields (Fig. 4).

This might have further implications for soil management in oil palm plantations at different maturity stages. Current practices for re-planting of the fields at the beginning of the next rotation cycle involve a removal and chipping of old oil palms and incorporation of approximately 20 to 50 t ha^−1^ of residues back into the soil (following recommendations by Kho & Jepsen, 2015), as well as the planting of cover crops to maintain soil fertility and limit soil erosion. However, our results indicate that these methods might be insufficient to compensate for SOC losses which are likely to be a result of high soil erosion rates. It is crucial to investigate processes by which SOC loss occurs at different maturity stages and to offer additional soil conservation measures to safeguard soil health throughout the length of a rotation. Mitigation of the SOC losses at the re-planting and early development stages can then shift the entire baseline to a higher overall SOC content and contribute to higher accumulation until SOC saturation is reached. Monitoring efforts focusing on the impact of plantation age on SOC content could be improved through the development of plantation age maps (e.g. by using methods by Jarayee et al. (2022) or Danylo et al. (2021) that integrate machine learning algorithms and satellite imagery) to establish the extent and year of establishment of oil palm plantations.

### Recommendations for future SOC monitoring efforts

Sampling design to monitor soil properties, especially dynamic indicators such as SOC content, should include baseline data and selection of a suitable interval between sampling campaigns, as well as the definition of an appropriate methodology that accounts for the bulk density (e.g. by applying equivalent soil mass instead of the fixed depth approach; Fowler et al., 2023) and the variation across sampling depths (Nayak et al., 2019). With the increased interest of private enterprises in monitoring of soil conditions across their land holdings, we provide several specific recommendations for future efforts involving large-scale soil sampling campaigns to improve the robustness of collected data and facilitate extrapolation of the findings through environmental modelling:

1. Use of existing data to inform monitoring efforts through a targeted ‘stratified’ approach to soil sampling, e.g. via application of environmental covariates to guide site selection or to facilitate digital soil mapping;
2. Development of stratified sampling design, e.g. similar number of sampled fields across represented regions and (in particular) age groups, will allow for stronger statistical inference (e.g. use of *space-for-time substitution* method);
3. Improvement of the sample bulking approach by decreasing the size of an area over which samples are combined and georeferencing individual sub-samples should be prioritised;
4. Strengthening of sampling design via the designation of within-field sampling zones to account for major differences in soil properties within individual fields, e.g. via collection of separate samples under canopy, under frond pile, in harvest path, etc.;
5. Determination of bulk density values for each sample to allow for SOC stock estimations and enable viable comparisons;
6. Evaluation of the potential application of existing digital technologies (e.g. near-infrared spectrometers) as more economically viable approaches to determining a wide range of soil properties;
7. Documentation of land-use and land-cover history for individual fields to improve analyses and inform the delineation of at-risk areas for SOC loss;
8. Development of thematic maps such as oil palm plantation age maps or maps showing the historical distribution of peatlands to inform more in-depth analysis of SOC changes;
9. Storing a subset of samples from previous sampling campaigns to assess the comparability of the laboratory results in the event of changes in soil testing methods between sampling periods (e.g. due to replacement of laboratory equipment).

## Supplementary Information

Below is the link to the electronic supplementary material.

Supplementary file1 (DOCX 376 KB)

Supplementary file2 (CSV 191 KB)

Supplementary file3 (R 27.1 KB)

Supplementary file4 (R 15.5 KB)

Supplementary file5 (R 16.7 KB)

## Data Availability

In response to the study ‘Reproducibility goes on trial in ecology’, both the dataset and the annotated R scripts (laying out the analytical choices) generated for this study will be made available via Zenodo (10.5281/zenodo.10869055) upon the paper’s publication.
