# Supplementary material for: The older, the better: a comprehensive survey of soil organic carbon under commercial oil palm plantations
Source: Environ Monit Assess. 2024 Dec 21;197(1):86. doi: 10.1007/s10661-024-13540-y (PMC11663155; doi:10.1007/s10661-024-13540-y)
Supplement: Supplementary file 1 — Supplementary file1 (DOCX 376 KB) [file 10661_2024_13540_MOESM1_ESM.docx]

Supplementary material

**Figure S1.** A positive relationship between soil organic carbon (in %) and cation exchange capacity (ccmol kg^-1^) (R^2^ = 0.45).


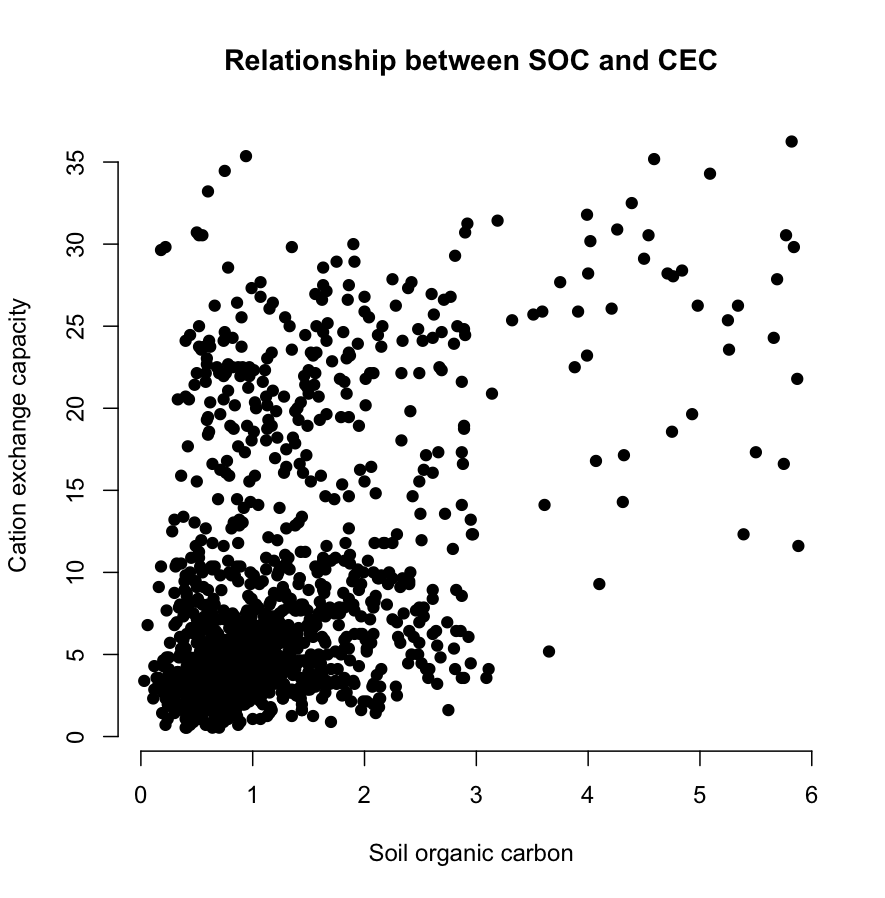


**Figure S2.** A Cleveland dotplot of soil pH (A, no unit) and SOC (B, unit: %) demonstrating outliers recorded for three distinct regions.
